# Biological clustering supports both “Dutch” and “British” hypotheses of asthma and chronic obstructive pulmonary disease

**DOI:** 10.1016/j.jaci.2014.06.035

**Published:** 2015-01

**Authors:** Michael A. Ghebre, Mona Bafadhel, Dhananjay Desai, Suzanne E. Cohen, Paul Newbold, Laura Rapley, Jo Woods, Paul Rugman, Ian D. Pavord, Chris Newby, Paul R. Burton, Richard D. May, Chris E. Brightling

**Affiliations:** a Department of Infection, Immunity and Inflammation, Institute for Lung Health, University of Leicester, Leicester, United Kingdom; b NIHR Respiratory Biomedical Research Unit, University Hospitals of Leicester, Leicester, United Kingdom; c Department of Health Sciences, University of Leicester, Leicester, United Kingdom; d Department of Respiratory Medicine, Nuffield Department of Clinical Medicine, University of Oxford, Headington, Oxford, United Kingdom; e MedImmune Ltd, Milstein Building, Granta Park, Cambridge, United Kingdom; f School of Social and Community Medicine, University of Bristol, Bristol, United Kingdom

**Keywords:** Asthma and COPD overlap, cytokines, factor and cluster analyses, COPD, Chronic obstructive pulmonary disease, ROC, Receiver operating characteristic, ROC AUC, Area under the receiver operating characteristic curve

## Abstract

**Background:**

Asthma and chronic obstructive pulmonary disease (COPD) are heterogeneous diseases.

**Objective:**

We sought to determine, in terms of their sputum cellular and mediator profiles, the extent to which they represent distinct or overlapping conditions supporting either the “British” or “Dutch” hypotheses of airway disease pathogenesis.

**Methods:**

We compared the clinical and physiological characteristics and sputum mediators between 86 subjects with severe asthma and 75 with moderate-to-severe COPD. Biological subgroups were determined using factor and cluster analyses on 18 sputum cytokines. The subgroups were validated on independent severe asthma (n = 166) and COPD (n = 58) cohorts. Two techniques were used to assign the validation subjects to subgroups: linear discriminant analysis, or the best identified discriminator (single cytokine) in combination with subject disease status (asthma or COPD).

**Results:**

Discriminant analysis distinguished severe asthma from COPD completely using a combination of clinical and biological variables. Factor and cluster analyses of the sputum cytokine profiles revealed 3 biological clusters: cluster 1: asthma predominant, eosinophilic, high T_H_2 cytokines; cluster 2: asthma and COPD overlap, neutrophilic; cluster 3: COPD predominant, mixed eosinophilic and neutrophilic. Validation subjects were classified into 3 subgroups using discriminant analysis, or disease status with a binary assessment of sputum IL-1β expression. Sputum cellular and cytokine profiles of the validation subgroups were similar to the subgroups from the test study.

**Conclusions:**

Sputum cytokine profiling can determine distinct and overlapping groups of subjects with asthma and COPD, supporting both the British and Dutch hypotheses. These findings may contribute to improved patient classification to enable stratified medicine.

Asthma and chronic obstructive pulmonary disease (COPD) cause considerable morbidity and consume substantial health care resources.^1,2^ Both conditions are characterized by airflow obstruction, which is typically variable and reversible in asthma but fixed in COPD. However, there is overlap; in severe asthma, there can be persistent airflow obstruction and partially reversible airflow obstruction in COPD. Likewise although some reports have suggested that there are marked differences in patterns of the underlying inflammation,^3^ cellular mechanisms, inflammatory mediators, and response to therapy^4^ between asthma and COPD, others have demonstrated considerable heterogeneity in severe asthma^5-7^ and COPD^8-11^ with overlap between the conditions.^12-14^ Indeed, there is an ongoing debate between the “Dutch hypothesis,” which proposes that asthma and COPD are manifestations of the same basic disease process, and the “British hypothesis,” which suggests that asthma and COPD are distinct entities generated by different mechanisms.^15^

The need to refocus efforts to define the similarities and differences in asthma, particularly in those with severe disease, and COPD in terms of cytokine profiles^16^ is underscored by the emergence of highly specific anti-inflammatory therapies because response is more likely to be phenotype rather than disease-specific.^17^ This is perhaps best exemplified by anti–IL-5 approaches, which have demonstrated clinical responses related to underlying eosinophilic lung inflammation in asthma^18,19^ and similar strategies are currently being tested in COPD. To enable these and further analogous developments, there is an urgent need to define the airway cytokine profiles in asthma and COPD. We hypothesized that there are distinct sputum cytokine profiles that are COPD and asthma specific and another that represents that asthma and COPD overlap.

## Methods

### Subjects

Subjects with severe asthma or moderate-to-severe COPD were recruited from a single center at the Glenfield Hospital, Leicester, United Kingdom, into independent test and validation studies. Assignment to asthma or COPD was made by the subjects' physician consistent with definitions of asthma and COPD according to the Global Initiative for Asthma^1^ or the Global Initiative for Chronic Obstructive Lung Disease^2^ guidelines, respectively, for both the test and validation groups. All subjects were assessed at stable visits at least 8 weeks free from an *exacerbation*, defined as an increase in symptoms necessitating a course of oral corticosteroids and/or antibiotic therapy. The subjects with COPD had participated in an exacerbation study,^20,21^ and some of the subjects with asthma had participated in an earlier study.^22^ All subjects provided written informed consent, and the studies were approved by the local Leicestershire, Northamptonshire, and Rutland ethics committee.

### Measurements

Demographic, clinical, and lung-function data were recorded including pre- and postbronchodilator FEV_1_, forced vital capacity, and symptom scores using the visual analogue scale. Spontaneous or induced sputum was collected for sputum total and differential cell counts and bacteriology; cell-free sputum supernatant was used for mediator assessment as described previously.^20^ Sputum was produced spontaneously in 93% of the subjects. Positive bacterial colonization was defined as colony-forming units greater than 10^7^/mL sputum or positive culture.^20,21^ Subjects with sputum eosinophil and neutrophil differential cell counts above 3%^23,24^ and 61%^25^ were defined as eosinophilic or neutrophilic, respectively. Further stratification of the subjects into 4 subgroups on the basis of their sputum cell counts was also done: pure eosinophilic (eosinophil > 3% and neutrophil < 61%), pure neutrophilic (eosinophil < 3% and neutrophil > 61%), mixed granulocytic (eosinophil > 3% and neutrophil > 61%), and paucigranulocytic (eosinophil < 3% and neutrophil < 61%). Inflammatory mediators were measured in sputum supernatants using the Meso Scale Discovery Platform (MSD; Gaithersburg, Md). The mediators measured were selected to reflect cytokines, chemokines, and proinflammatory mediators implicated in airway disease. The performance of the MSD platform in terms of recovery of spiked exogenous recombinant proteins has been described previously.^16^ Sputum inflammatory mediators that were below the detectable range were replaced with their corresponding lower limit of detection in subjects with both asthma and COPD. Twenty-one mediators were included in the test study, and 14 mediators were available in the validation study.

### Statistical analysis

See this article's Online Repository at www.jacionline.org for detailed statistical methods. All statistical analyses were performed using STATA/IC version 13.0 for Windows (StataCorp, College Station, Tex) and R version 2.15.1 (R Foundation for statistical computing, Vienna, Austria). Parametric data were presented as mean with SEM, and log transformed data were presented as geometric mean with 95% CI. The χ^2^ test or the Fisher exact test was used to compare proportions, and 1-way ANOVA was used to compare means across multiple groups; nonparametric data were presented as median with first and third quartiles, and Kruskal-Wallis test was used to compare subgroups. Inflammatory mediators that significantly discriminated across asthma versus COPD and bacterially colonized versus noncolonized were identified using receiver operating characteristic (ROC) curves. Factor analysis was performed on sputum inflammatory mediators, and independent factor scores were derived and used as input variables in the k-means cluster analysis to identify subjects' biological subgroups. The optimal number of clusters was chosen on the basis of a scree plot, by plotting within-cluster sum of the squares against a series of sequential numbers of clusters. Linear discriminant analysis was performed and a classification model developed from the test study for the validation study. In addition, classification and regression trees analysis was performed sequentially on all inflammatory mediators in the test study that had high discriminant function to identify possibly clinically relevant cutoff points. The inflammatory mediator cutoff points with the highest sensitivity ratio in discriminating the clusters together with subject disease status (asthma or COPD) were applied to classify the validation study into subgroups. A *P* value of less than .05 was taken as statistically significant.

## Results

**Fig E1:**
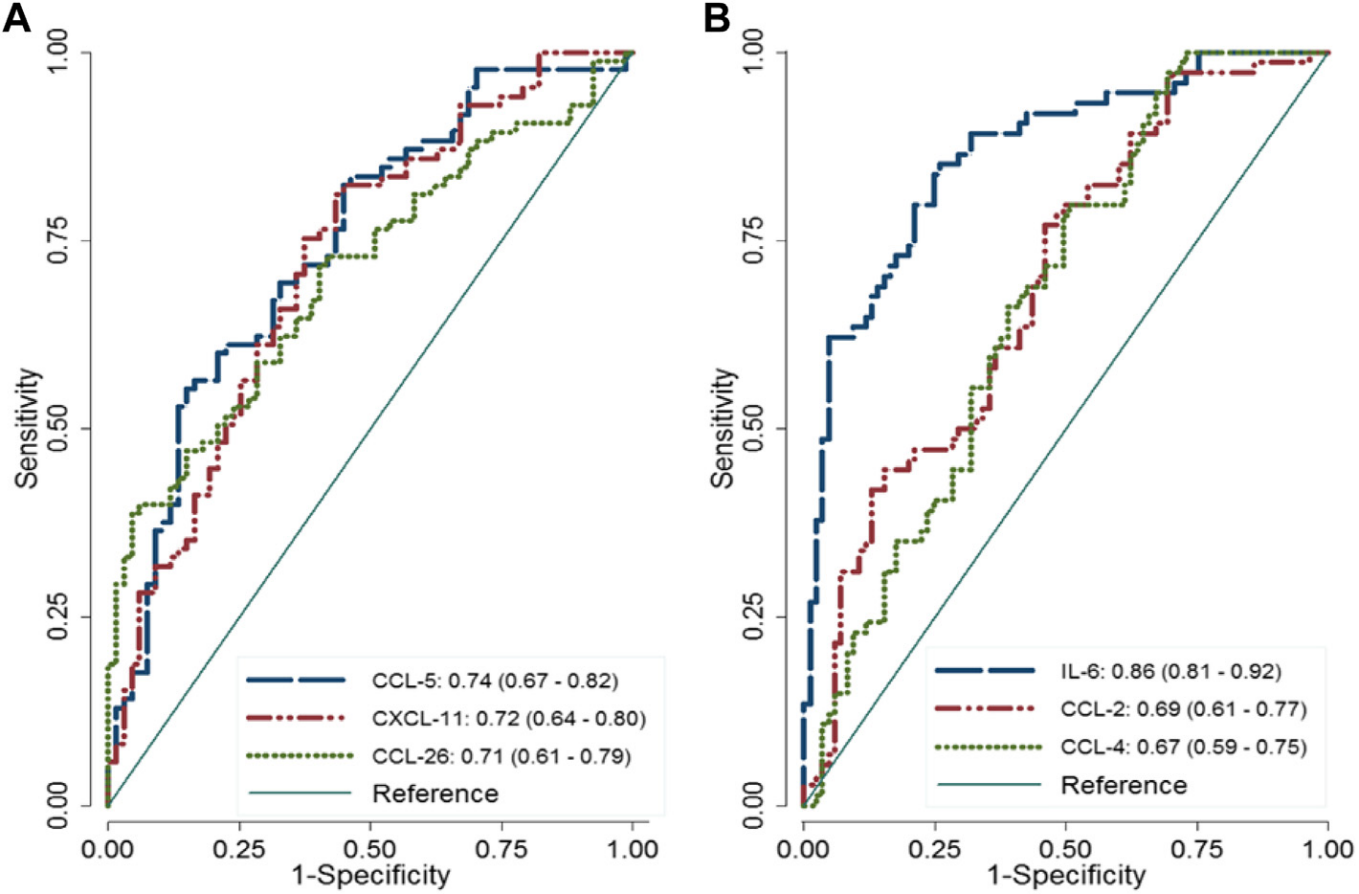
Sputum cytokines, which discriminated across asthma and COPD. ROC AUC with 95% CI illustrating biomarkers that predict asthma **(A)** or COPD **(B)**.

**Fig 1:**
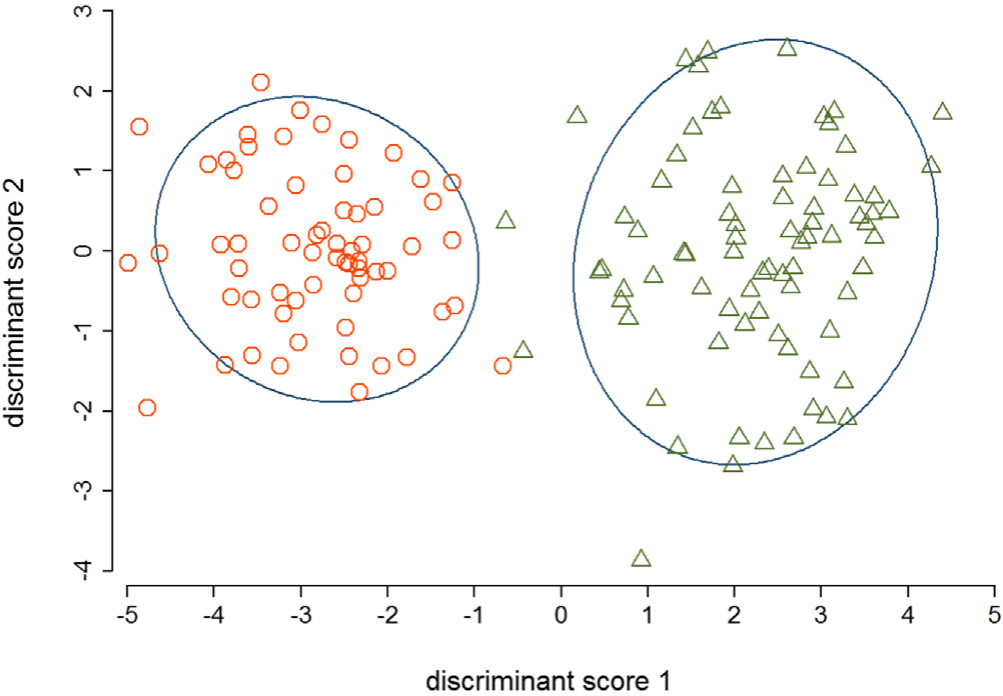
Discriminant function of demographic, clinical, lung function, and sputum cytokines characteristics across asthma and COPD. *Hollow triangles* indicate asthma and *hollow circles* indicate COPD.

The clinical and sputum characteristics of the asthma (n = 86) and COPD (n = 75) test groups are presented in Table I. Subjects with asthma were younger, had a higher body mass index, better lung function, fewer symptoms, and a lower smoking pack-year history than did subjects with COPD. The differential neutrophil and eosinophil counts were not statistically different between the groups, but the total cell count was higher in those with COPD. The sputum inflammatory mediator profiles were distinct with increased T_H_2 (IL-5, IL-13, and CCL26) and T_H_1 mediators (CXCL10 and 11) in severe asthma compared with COPD and increased IL-6, CCL2, CCL3, and CCL4 in COPD compared with severe asthma. Inflammatory mediators that best discriminated asthma and COPD are presented as ROC curves (see Fig E1 in this article's Online Repository at www.jacionline.org). Sputum CCL5 and CXCL11 levels were substantially higher in subjects with asthma than in subjects with COPD, with area under the ROC curves (ROC AUCs) of 0.74 (95% CI, 0.67-0.82; *P* < .0001) and 0.72 (95% CI, 0.64-0.80; *P* < .0001), respectively. Sputum IL-6 and CCL2 levels were significantly higher in subjects with COPD than in subjects with asthma, with ROC AUCs of 0.86 (95% CI, 0.81-0.92; *P* < .0001) and 0.69 (0.61-0.77; *P* < .0001), respectively. Discriminant analysis using the combined clinical, physiological, and biological (inflammatory mediator) variables completely distinguished the asthma and COPD groups (Fig 1).

**Fig E2:**
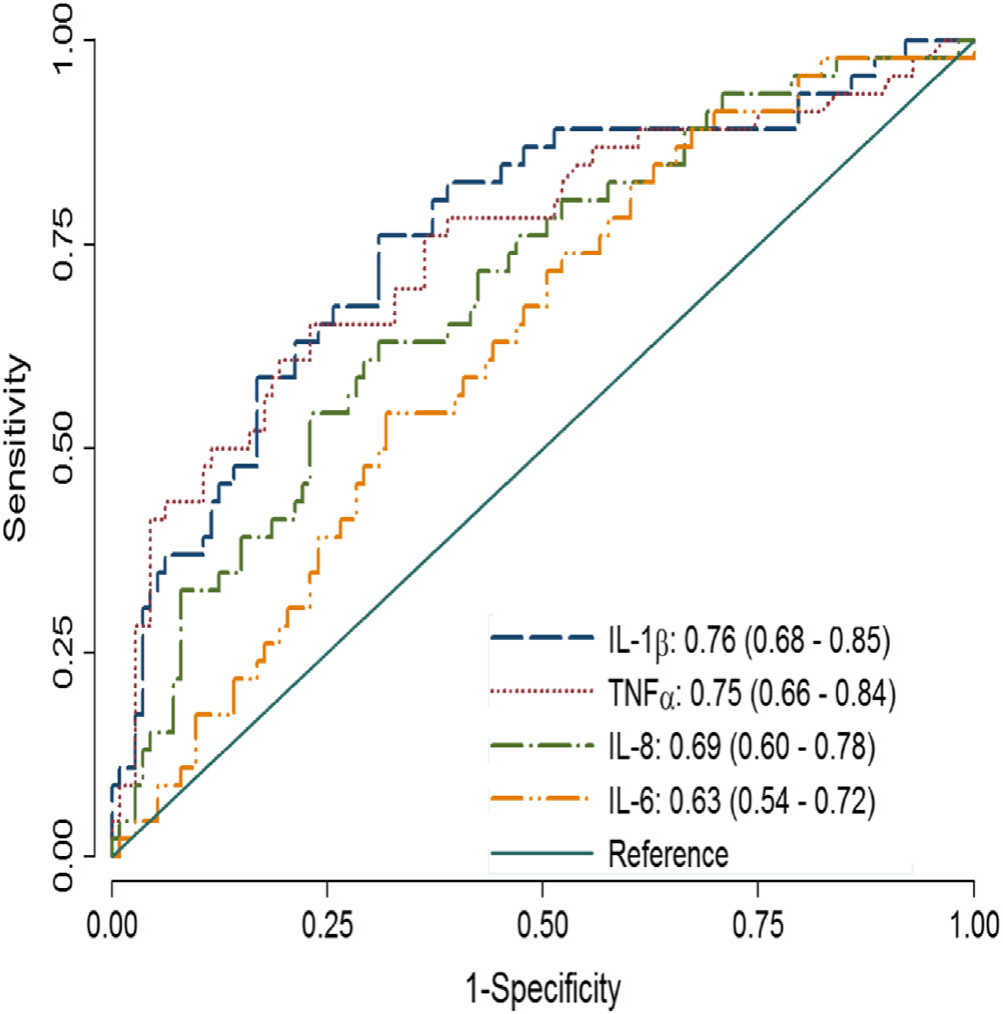
Cytokines across bacterial colonization and low-bacterial colonization. ROC AUC with 95% CI illustrating biomarkers that predict bacterial colonization.

**Fig 2:**
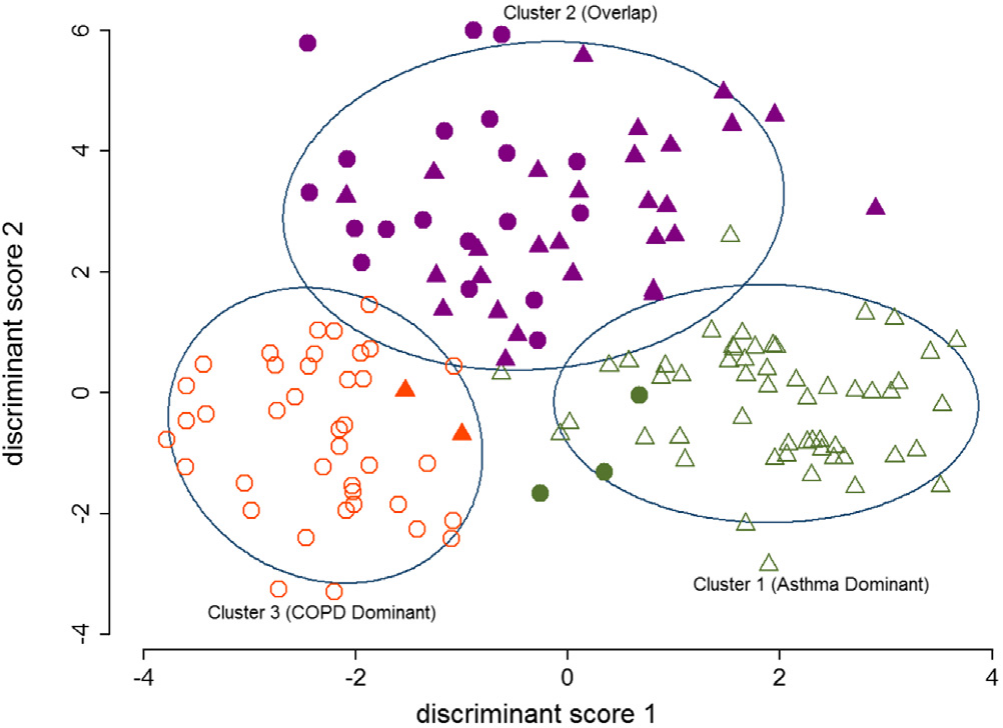
The 3 identified biological clusters presented using subjects' discriminant scores. *Hollow triangles* indicate eosinophilic asthma dominant (95% asthma, n = 58); *bold triangle* and *bold circle*, neutrophilic asthma and COPD (overlap) dominant (59.6% asthma, n = 47); *hollow circle*, COPD dominant (95% COPD, n = 41); *bold triangle*, overlapped asthma; *bold circle*, overlapped COPD.

The mediators that best discriminated between bacterially colonized and noncolonized subjects were sputum IL-1β and TNF-α, with ROC AUCs of 0.76 (95% CI, 0.68-0.85) and 0.75 (95% CI, 0.66-0.84), respectively (see Fig E2 in this article's Online Repository at www.jacionline.org). Factor analysis revealed 4 factors with IL-1β, IL-5, IL-6, and CXCL11 as the highest loading components, respectively, across the 4 factors (see Table E1 in this article's Online Repository at www.jacionline.org). Subsequent cluster analysis identified 3 clusters (Table II). Individual clinical and biological comparisons of subjects with asthma and COPD in clusters 1, 2, and 3 are presented in Table E2 in this article's Online Repository at www.jacionline.org. Linear discriminant analysis was performed to verify the determined clusters and to identify the contribution of inflammatory mediators in discriminating the clusters. Subsequently, 2 discriminant scores for individual subjects were calculated and used to represent the clusters in a 2-dimensional graph (Fig 2).

Cluster 1 consisted of mainly subjects with asthma (95% of cluster 1) with elevated sputum T_H_2 mediators and was eosinophil predominant, with 67% of the subjects having a sputum eosinophilia and 48% a sputum neutrophilia. Further stratification of cluster 1 by sputum cell counts showed that the subjects were 40% pure eosinophilic, 21% pure neutrophilic, 27% mixed granulocytic, and 12% paucigranulocytic.

Cluster 2 consisted of an overlap of subjects with asthma and COPD with sputum neutrophil predominance (75% of subjects with asthma and 95% of subjects with COPD). In contrast, only 11% and 5% of the subjects with asthma or COPD, respectively, had a sputum eosinophilia. In addition, there were elevated sputum levels of IL-1β, IL-8, IL-10 and TNF-α and bacterial colonization. The increased rate of bacterial colonization found in this cluster was driven predominately by subjects with COPD (Table E2). Further stratification of cluster 2 showed that the subjects were 0% pure eosinophilic, 74% pure neutrophilic, 9% mixed granulocytic, and 17% paucigranulocytic.

Cluster 3 consisted predominantly of subjects with COPD (95% of cluster 3). In contrast to subjects with COPD in cluster 2, neutrophilic inflammation was present in only 49% of the subjects whereas a sputum eosinophilia was observed in 46% of the subjects. IL-6 and CCL2 levels were increased compared with those in clusters 1 and 2 but were similar to those in subjects with COPD in cluster 2. Only CCL13 and CCL17 were elevated in subjects with COPD in cluster 3 compared with subjects with COPD in cluster 2 (Table E2). Further stratification of cluster 3 showed that the subjects were 21% pure eosinophilic, 28% pure neutrophilic, 23% mixed granulocytic, and 28% paucigranulocytic. The proportion of subjects with asthma with airflow obstruction in the 3 clusters was not significantly different. Of the 2 subjects with asthma in cluster 3, 1 had persistent airflow obstruction compared with 17 of 55 in cluster 1 and 10 of 28 in cluster 2.

**Fig E3:**
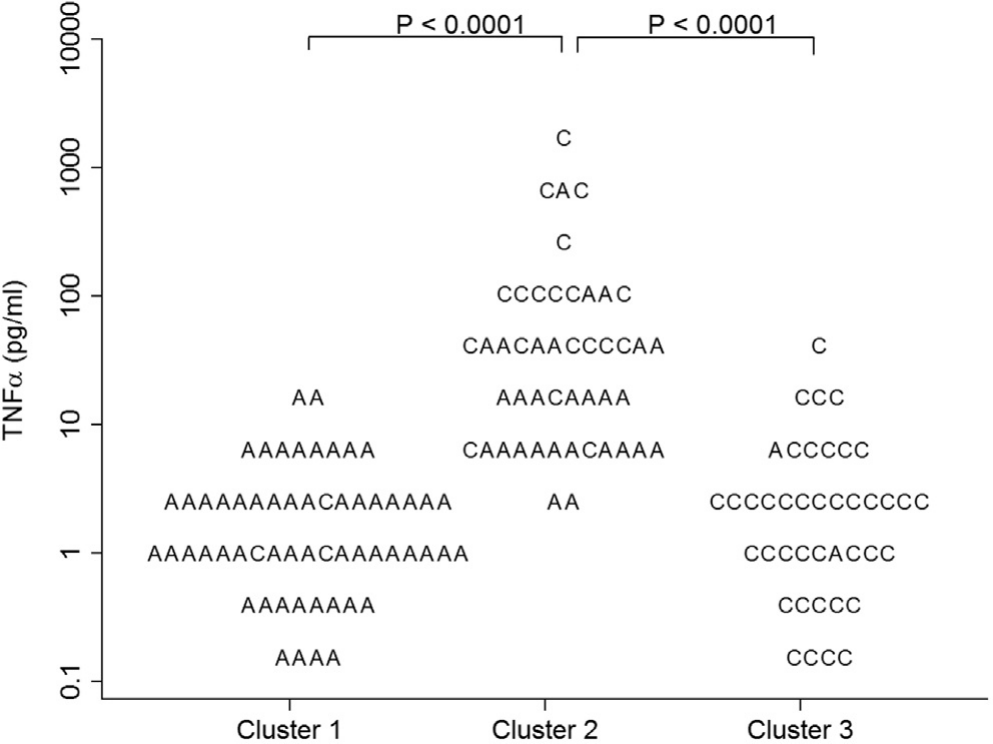
Absolute TNF-α concentrations on a log scale (base 10) across the 3 identified biological clusters. *A*, Asthma; *C*, COPD. *P* is the *P* value for mean comparison between cluster 1 or cluster 3 versus cluster 2 (overlap).

**Fig 3:**
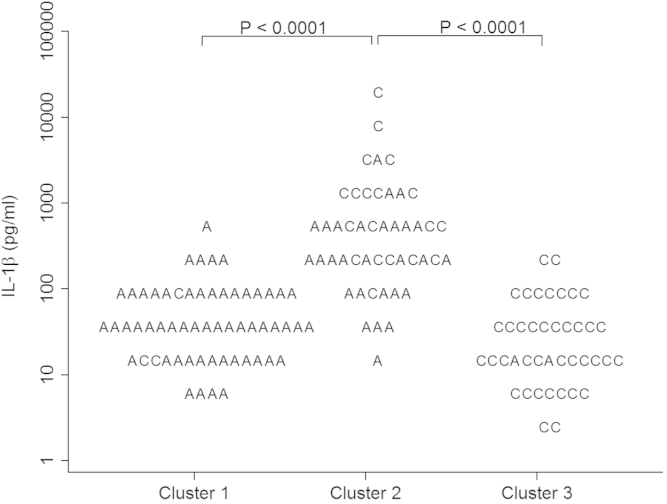
Absolute IL-1β concentrations on a log scale (base 10) across the 3 identified biological clusters. *A*, Asthma; *C*, COPD. *P* is the *P* value for mean comparison between cluster 1 or cluster 3 versus cluster 2 (overlap).

The best discriminator between subjects in clusters 1 or 3 compared with the overlap group cluster 2 was sputum IL-1β at a cutoff point of 130 pg/mL (Fig 3). The second best discriminator was TNF-α with a cutoff point of 5 pg/mL (see Fig E3 in this article's Online Repository at www.jacionline.org).

### Validation

**Fig 4:**
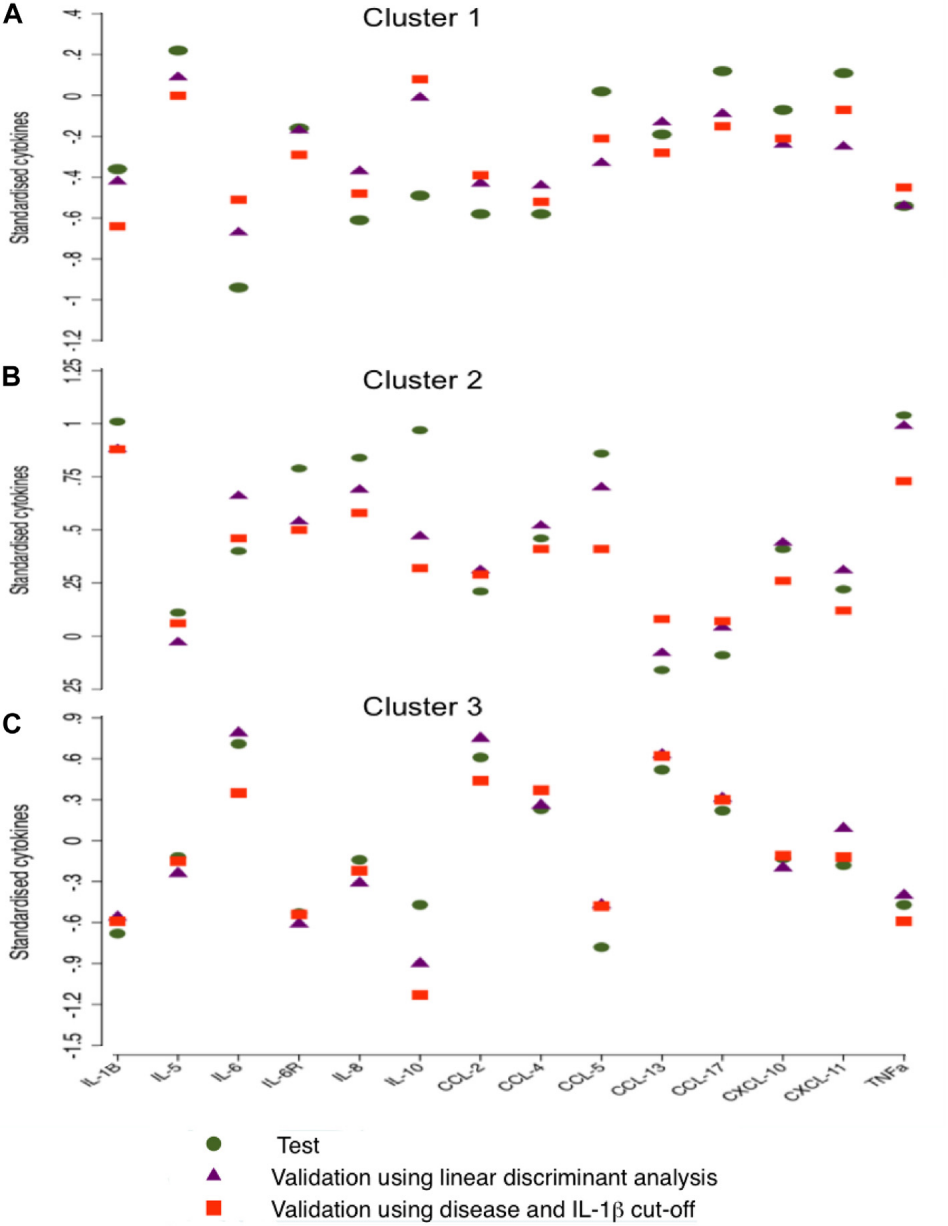
Cytokine profiles in the test and the validation groups using linear discriminant analysis or IL-1β cutoff and disease for cluster 1 **(A)**, cluster 2 **(B)**, and cluster 3 **(C)**. *Circles* indicate test study, *triangles* indicate validation using linear discriminant analysis, and *rectangles* indicate validation using IL-1β cutoff at 130 pg/mL and disease status (asthma or COPD). The *y-axes* depict the mean *z* value (standardized) of each cytokine in each test and validation subgroup.

These cluster analysis findings were then validated in independent asthma and COPD cohorts. Subjects were assigned into subgroups using 2 techniques. The first was a classification model developed from the test cohort using linear discriminant analysis and betas for each cluster and cytokines were extracted (see Table E3 in this article's Online Repository at www.jacionline.org). Individual subject discriminant score in each subgroup was calculated and the subject was assigned to the subgroup in which he or she had the highest score. The second technique used the IL-1β cutoff point at 130 pg/mL, which was identified as the best classifier to distinguish overlap cluster 2 from clusters 1 or 3 in the test study and was used alongside subject disease status (asthma or COPD). The sputum cellular and inflammatory mediator profiles of the 3 validation study subgroups, obtained using both techniques, were very similar to the test subgroups (Tables III and IV; Fig 4). In addition, individual clinical and biological comparisons of subjects with asthma and COPD in validation subgroups, presented in this article's Online Repository in Tables E4 and E5 at www.jacionline.org, revealed a patter similar to that of test subgroups (Table E2).

## Discussion

Here we report that although a combination of clinical variables distinguished asthma from COPD, further analyses of the sputum inflammatory mediators revealed that patients with asthma and COPD were best described by 3 biological clusters incorporating clinical, physiological, and inflammatory mediator characteristics. Our findings have further underscored the complex heterogeneity of asthma and COPD and provided support for the “British” hypothesis of airway disease pathogenesis as we identified 2 clusters that were predominately either asthma or COPD with distinct cytokine profiles, while also supporting the “Dutch” hypothesis by identifying a third cluster of overlapping subjects from both disease groups with similar cytokine profiles. Cluster 1 was asthma predominant with evidence of eosinophilic inflammation and increased T_H_2 inflammatory mediators. Cluster 2 contained an asthma and COPD overlap group, with predominately neutrophilic airway inflammation and elevated levels of IL-1β and TNF-α in addition to being assigned the highest proportion of subjects with bacterial colonization. Cluster 3 was a COPD-predominant group with mixed granulocytic airway inflammation and high sputum IL-6 and CCL13 levels. Furthermore, the biological clusters derived from the test group could be validated in an independent group yielding similar inflammatory mediator profiles to the test group. Whether these biological clusters can be used to stratify subjects for more targeted approaches to novel and existing therapies needs to be further studied.

The clusters we have identified have biological plausibility and they confirm and extend our current understanding of the immunopathobiology of asthma and COPD, moving our understanding beyond previous comparisons of asthma versus COPD^16^ or clustering approaches of cytokine profiles in asthma or COPD alone.^20,26^ In addition, the clusters might represent groups with possible stratified responses to specific anti-inflammatory treatment. Cluster 1 is consistent with the T_H_2-predominant eosinophilic asthma paradigm. Indeed, this group was predominately asthmatic but importantly also included about 5% of subjects with COPD. It would seem likely that this group is most likely to respond to anti-T_H_2 cytokine therapy such as anti–IL-5 and 13.^18,19,27,28^ Eosinophilic COPD is well-described, and this group has a greater response to oral and inhaled corticosteroids than did those with noneosinophilic COPD.^29,30^ Whether subjects with COPD in this cluster would respond to anti-T_H_2 cytokine therapy is currently under study (www.clinicaltrials.gov NCT01227278). Cluster 2 included an overlap of subjects with asthma and COPD. This group was predominately neutrophilic, consistent with previous observations,^14^ and with increased bacterial colonization. Recent evidence supports the role for macrolide antibiotics in COPD^31^ and in noneosinophilic severe asthma.^32^ Antineutrophilic strategies such as anti-CXCR2 are currently under study.^33^ Further studies are required to assess whether this cluster represents patients most likely to respond to these therapies. In cluster 2, increased bacterial colonization was evident, particularly in those with COPD, perhaps suggesting that in these subjects the neutrophilic inflammation is a consequence of bacterial colonization rather than the primary abnormality. Thus, whether ameliorating neutrophilic inflammation in this group is beneficial or harmful is unclear. Indeed, lessons from anti–TNF-α therapy suggest that targeting proinflammatory cytokines can increase the risk of infection.^34,35^ In contrast, in those with neutrophilic inflammation without evidence of bacterial colonization, particularly in those with asthma, the neutrophilic inflammation might be critical in the development of the disease. Thus, identification of distinct groups that benefit or are harmed by antineutrophilic approaches would enable better stratification of such therapies. Cluster 3 included mainly subjects with COPD in which bacterial colonization was observed in fewer subjects in spite of consistently elevated proinflammatory cytokines. Perhaps this group, in contrast to cluster 2, represents subjects in which the proinflammatory environment plays a more causal role in the disease expression rather than as a consequence of infection. This might suggest that this group would be more amenable to anticytokine therapies such as anti–IL-6. In addition, eosinophilic inflammation was a feature in some subjects in cluster 3 in the absence of an elevated T_H_2 profile. One of the few cytokines increased in cluster 3 was CCL13, which is a CCR3 agonist and promotes eosinophil migration. Small airway macrophages are an important source of CCL13 in the airway and might play a role in the eosinophilic inflammation in this group.^36^ Taken together, these intriguing and novel observations immediately open up opportunities for further translational studies to determine the underlying mechanisms of these clusters and their treatment-specific anti-inflammatory therapies.

In addition to clear differences in the cytokine profiles between groups, there were several differences in clinical parameters. These were largely dependent on whether the clusters were asthma or COPD predominant or mixed. For example, lung function, age, greater smoking history, and higher exacerbation frequency was related to the number of subjects with COPD in the cluster. However, the symptom of cough was more common in cluster 2. Indeed, subjects with asthma and COPD in cluster 2 had a higher visual analog scale score for cough than did either the subjects with asthma in cluster 1 or the subjects with COPD in cluster 3, respectively. This suggests that this difference is independent of disease status and might represent a real association either between the inflammatory profile or increased bacterial colonization in cluster 2. This cluster also had the highest sputum total inflammatory cell count, suggesting that this represents a “chronic bronchitis” group. As suggested above, whether this group might warrant antimicrobial, anti-inflammatory, or antimucolytic therapy is an interesting possibility.

One of the strengths of our observations is that we were able to support the identification of the 3 biological clusters in an independent validation group. The similarity between the cytokine (inflammatory mediators) profiles in test and validation groups supports the view that each cluster is a consistent phenotype and might reflect common immunopathology and phenotype-specific responses to treatment. We found biological clusters that were asthma or COPD predominant, suggesting that there are distinct mechanisms underlying these groups, but we also identified a consistent overlap group that might be a consequence of shared mechanisms. Two approaches were used to validate the clusters in an independent group using discriminant analysis and the generation of a classifier that used the disease allocation and sputum IL1β cutoff. Sputum IL-1β was the best discriminator between the subjects with asthma or COPD in clusters 1 and 3, respectively, with those in the overlap group cluster 2. The clinical diagnosis of asthma or COPD together with a single sputum cytokine (IL-1β cutoff) demonstrated a simple approach to segment asthma and COPD populations into 3 groups with distinct and consistent cytokine profiles. This approach has advantages in its simplicity and offers the potential for immediate use in stratified medicine studies although it might underestimate small, albeit potentially important subgroups such as T_H_2 high COPD.

One possible limitation of this study is that only subjects with severe asthma and COPD who attended a secondary care setting were included, and thus might not be representative of a more generalized population. We concede that our findings cannot be extrapolated to mild to moderate asthma or mild COPD but are confident that our test and validation populations are representative of our broader secondary care patient population. Our earlier preliminary data comparing asthma and COPD included subjects across the severity of disease and in this analysis fewer differences between asthma and COPD were observed.^16^ Whether this was due to lack of power because of the small numbers or due to masking clearer differences in more severe disease is unknown. Further studies are required to include healthy controls, larger disease populations including a broader spectrum of subjects including those with mild disease, and comparisons with other disease control groups. Allergic sensitization might also be an important mechanism in driving the different clusters. We did not record atopic status in the COPD group consistently, but in those with asthma there was no difference across the clusters. However, future studies should consider the role of allergy in these clusters. Our study has focused on stable visits and a similar comparison is required for longitudinal follow-up at stable and exacerbation events. We have previously reported exacerbation biological clusters in subjects with COPD and interestingly have identified similar profiles as described here.^20^ Whether comparisons of cytokine profiles in larger groups of subjects with severe asthma and COPD reveal similar biological clusters needs to be addressed. The cytokine profiles have been derived from sputum analysis and whether the profiles are similar in tissue samples is unknown. Access to bronchoscopic samples from large numbers of subjects with COPD and asthma with severe disease is challenging, but multicenter efforts to address this are underway in parallel with sputum sampling and these findings are eagerly awaited. In addition, although we have chosen to measure a large number of mediators implicated in obstructive airway disease, these mediators cannot fully reflect the complexity of airway disease and approaches using more comprehensive assessment of inflammatory networks in the airway perhaps using 'omic approaches such as transcriptomics will be informative.^37^ Such studies in small numbers suggest similar groupings described here with transcriptional profiles associated with cellular profiles and further studies are awaited.

In conclusion, we found here that sputum inflammatory mediator profiling can determine distinct and overlapping groups of subjects with asthma and COPD. We identified an asthma-predominant cluster with eosinophilic inflammation and elevated T_H_2 inflammatory mediators, a COPD-predominant group with elevated proinflammatory cytokines, and an asthma and COPD overlap group that clinically had chronic bronchitis, increased bacterial colonization, elevated sputum IL-1β and TNF-α levels, and a sputum neutrophilia. We predict that these groups might contribute to improved patient classification to enable a stratified medicine approach to airways disease.

**Clinical implications**

Sputum cytokine profiling can determine distinct and overlapping asthma and COPD subgroups supporting both the British and Dutch hypotheses of airway disease.

## Figures and Tables

**Table E1:** Factor loading matrix for sputum cytokines after orthogonal varimax rotation

Variable	Factor loadings				C1	U2
	Factor 1	Factor 2	Factor 3	Factor 4		
IL-1β	**0.93**				088	0.12
IL-5		**0.84**			0.78	0.22
IL-6			**0.81**		0.75	0.25
IL-6R	0.73				0.65	0.35
IL-8	0.73		0.50		0.82	0.18
IL-13		0.73			0.56	0.44
CCL-2			0.70		0.55	0.45
CCL-3	0.49		0.56		0.69	0.31
CCL-4			0.59		0.66	0.34
CCL-5	0.65			0.54	0.79	0.21
CCL-13			0.64		0.65	0.35
CCL-17		0.74			0.72	0.28
CCL-26		0.75			0.63	0.37
CXCL-11				**0.74**	0.64	0.36
TNF-α	0.88				0.82	0.18
VEGF	0.57				0.54	0.46
Eigenvalue	4.05	3.05	2.92	1.12		
Pr (%)	35.4	26.7	25.5	9.8		

Factor loading less than 0.45 were replaced with blank, but included when estimating the factor scores. Highest loading variables for each factor are in boldface. *C1*, Proportion of total variation accounted for by the common factors (common variance); *Pr*, percent of total variation accounted for by each factor; *U2*, proportion of total variation not accounted by the common factors (unique variance); *VEGF*, vascular endothelial growth factor.

**Table E2:** Summary statistics by disease and biological subgroups in the test study

Variable	Cluster 1	Cluster 2		Cluster 3	Pairwise comparison, *P* value		
	G1 (asthma in cluster 1) (n = 55)	G2 (asthma in cluster 2) (n = 28)	G3 (COPD in cluster 2) (n = 19)	G4 (COPD in cluster 3) (n = 39)	G1 vs G2	G2 vs G3	G3 vs G4
Males, n (%)	29 (52.7)	13 (46.4)	13 (68.4)	27 (69.2)	.59	.14	.95
Current or ex-smokers, n (%)	19 (34.5)	11 (39.3)	18 (94.7)	38 (97.4)	.67	.023	.6
Pack-year history∗	5.5 (3.1-9.8)	2.8 (1.3-5.8)	36.6 (27.8-48.1)	42.1 (35.3-50.2)	.16	<.0001	.4
Age (y)†	54 (1.6)	53 (2.4)	70 (2)	68 (1.7)	.65	<.0001	.36
Duration of disease (y)	24 (18.2-31.38)	15 (8.84-24.19)	4 (2.57-6.68)	5 (3.71-7.04)	.07	.001	.48
BMI (kg/m^2^)†	30.1 (1)	31.0 (1.3)	25.5 (1)	25.1 (0.7)	.61	.004	.72
Exacerbation number of steroids	3 (0.3)	3 (0.4)	4 (0.59)	4.59 (0.41)	.96	.26	.33
Maintenance prednisolone dose use, n (%)	34 (61.8)	16 (57.1)	3 (15.8)	5 (12.8)	.68	.006	.38
Daily prednisolone dose (mg)‡§	10 (10-15)	8.75 (5-10)	5 (4-5)	5 (5-5)	.044	.13	.3
Daily inhaled corticosteroid dose (μg/d)‡||	2000 (1000-2000)	1400 (1000-2000)	1000 (400-2000)	2000 (800-2000)	.056	.31	.25
Pre-FEV_1_ (L)†	2.21 (0.1)	2.06 (0.2)	1.27 (0.1)	1.27 (0.1)	.4	.001	.97
Pre-FEV_1_/FVC ratio (%)†	69.6 (1.9)	63.8 (2.7)	50.6 (3.4)	48.6 (2.4)	.08	.004	.63
Pre-FEV_1_ predicted (%)†	77.8 (2.8)	69.1 (4.5)	45.6 (4.9)	45.5 (3)	.087	.001	.97
Post-FEV_1_ (L)†	2.38 (0.1)	2.25 (0.16)	1.29 (0.14)	1.33 (0.09)	.5	<.001	.8
Post-FEV_1_ predicted (%)†	82.9 (2.8)	74.7 (4.7)	47.1 (4.8)	47.4 (2.9)	.11	<.0001	.96
Sputum neutrophil count (%)†	59.4 (3.2)	70.1 (4.4)	87.6 (2.4)	58.7 (3.3)	.052	.003	<.0001
Sputum eosinophil count (%)	3.9 (2.4-6.5)	0.7 (0.4-1.1)	0.6 (0.4-1.0)	2.3 (1.4-3.6)	<.0001	.89	.001
Sputum macrophage count (%)	15.9 (11.9-21.3)	17.5 (12.3-24.9)	7.2 (5.1-10.1)	25.6 (20.9-31.2)	.7	.001	<.0001
TCC (×10^6^ cells/g sputum)	1.28 (0.92-1.8)	2.62 (1.8-3.8)	10.4 (7.0-15.2)	1.8 (1.3-2.6)	.01	<.0001	<.0001
Blood eosinophil × 10^9^/L	0.23 (0.17-0.31)	0.27 (0.2-0.38)	0.23 (0.17-0.31)	0.22 (0.17-0.28)	.51	.45	.9
Blood neutrophil × 10^9^/L†	5.78 (0.3)	5.58 (0.4)	6.17 (0.6)	5.55 (0.4)	.68	.39	.35
CFU >10^7^/mL or positive culture, n (%)	7 (12.7)	9 (32.1)	17 (89.5)	9 (23.1)	.034	<.0001	<.0001
VAS score-cough (mm)†	30 (3.1)	42 (5.2)	57 (5.9)	37 (4.6)	.046	.061	.011
VAS score-dyspnea (mm)†	31 (3.5)	39 (4.2)	56 (4.3)	44 (4.6)	.13	.011	.11
IL-1β (pg/mL)	40.6 (31.4-52.5)	228.6 (148.7-351.5)	800.9 (444.8-1442)	23.9 (17.2-33.3)	<.0001	.001	<.0001
IL-4 (pg/mL)‡	0.36 (0.36-0.59)	0.36 (0.36-0.59)	0.36 (0.36-0.36)	0.36 (0.36-0.36)	.89	.41	.5
Detectable IL-4, n (%)	18 (32.7)	10 (35.7)	3 (15.8)	2 (5.1)	.79	.19	.32
IL-5 (pg/mL)	2.7 (1.7-4.5)	2.9 (1.6-5.4)	1.4 (0.7-2.7)	1.5 (0.9-2.3)	.88	.12	.87
IL-6 (pg/mL)	20.8 (14.4-29.9)	165.4 (113.4-241.3)	563.1 (342.6-925.5)	524.3 (351.3-782.5)	<.0001	<.0001	.84
IL-6R (pg/mL)	174.4 (134.6-226.0)	469.4 (338.4-651.2)	385.2 (283.8-522.9)	108.2 (84.8-138.1)	<.0001	.41	<.0001
IL-8 (pg/mL)	1727 (1242-2401)	10,225 (7,320-14,283)	11,934 (9,260-15,379)	3116 (2217-4379)	<.0001	.51	<.0001
IL-10 (pg/mL)	0.34 (0.25-0.47)	3.4 (1.9-6.1)	11.5 (6.4-20.6)	0.3 (0.2-0.6)	<.0001	.008	<.0001
IL-13 (pg/mL)	10.4 (7.6-14.2)	5.3 (3.9-7.2)	4.2 (2.8-6.4)	3.6 (2.4-5.3)	.009	.38	.61
IL-17 (pg/mL)‡	2.12 (2.12-4.27)	3.44 (2.12-10.2)	2.12 (2.12-2.12)	2.12 (2.12-2.12)	.033	.002	.52
Detectable IL-17, n (%)	20 (36.4)	17 (60.7)	2 (10.5)	0 (0.0)	.035	.001	.1
CCL-2 (pg/mL)	202 (161.3-252.9)	499.9 (327.7-762.8)	488.9 (377.3-633.4)	744.61 (519.0-1068.3)	<.0001	.94	.14
CCL-3 (pg/mL)	19.3 (14.0-26.5)	80.3 (52.8-122.0)	129.5 (84.2-199.3)	51.2 (38.4-68.3)	<.0001	.14	.001
CCL-4 (pg/mL)	228.2 (138.3-376.8)	885.5 (586.6-1336.7)	1648.0 (1157.7-2346.1)	846.7 (641.1-1118.3)	.001	.041	.007
CCL-5 (pg/mL)	5.8 (4.6-7.2)	20.9 (14.3-30.8)	9.0 (6.1-13.4)	2.2 (1.7-2.8)	<.0001	.006	<.0001
CCL-13 (pg/mL)	18.2 (12.7-26.1)	21.4 (14.6-31.4)	15.7 (8.7-28.2)	45.4 (34.1-60.4)	.58	.37	.001
CCL-17 (pg/mL)	27.3 (19.2-39.0)	26.1 (15.7-43.2)	14.4 (8.2-25.2)	33.6 (23.5-47.9)	.88	.14	.012
CCL-26 (pg/mL)	12.8 (7.9-20.8)	7.1 (4.2-12.1)	3.0 (1.7-5.3)	3.2 (2.0-5.0)	.15	.037	.89
CXCL-10 (pg/mL)	428.2 (288.6-635.3)	2002.4 (1359.3-2949.7)	247.6 (118.1-518.7)	362.5 (246.5-533.0)	<.0001	<.0001	.33
CXCL-11 (pg/mL)	35.3 (23.2-53.6)	149.9 (81.2-276.9)	6.6 (2.0-22.1)	18.3 (11.5-29.2)	<.0001	<.0001	.067
TNF-α (pg/mL)	1.4 (1.1-1.9)	16.0 (10.1-25.3)	75.1 (40.1-140.5)	1.6 (1.0-2.5)	<.0001	<.0001	<.0001
VEGF (pg/mL)	1056 (885-1262)	2523 (2011-3166)	1796 (1486-2169)	1209 (1011-1447)	<.0001	.041	.01
IFN-γ (pg/mL)‡	0.13 (0.13-0.13)	0.18 (0.13-1.37)	0.39 (0.13-2.05)	0.13 (0.13-0.16)	.003	.91	.044
Detectable IFN-γ, n (%)	9 (16.4)	14 (50)	10 (52.6)	11 (28.2)	.001	.99	.07

Data presented as geometric mean (95% CI) unless stated. *BMI*, Body mass index; *FVC*, forced vital capacity; *TCC*, total sputum cell count; *VAS*, visual analog scale; *VEGF*, vascular endothelial growth factor.

∗ Pack-year history of current and ex-smokers.

† Mean (SEM).

‡ Median (first and third quartiles).

§ Dose for only those subjects prescribed daily prednisolone.

|| Beclomethasonedipropionate equivalent.

**Table E3:** Coefficients (βs) and class proportion in each cluster in the test study used to predict class membership in the validation study

Variables	Cluster 1 (*β*_1k_)	Cluster 2 (*β*_2k_)	Cluster 3 (*β*_3k_)
IL-1B	1.63	2.17	0.96
IL-5	−6.31	−6.50	−6.79
IL-6	−2.62	−1.17	0.14
IL-6R	6.28	5.58	4.72
IL-8	6.64	7.59	7.31
CCL-2	5.56	5.66	6.98
CCL-4	3.59	3.83	4.66
CCL-5	−1.36	−0.80	−2.83
CCL-13	−0.04	−0.49	−0.21
CCL-17	2.13	1.09	1.09
CXCL-11	0.48	0.51	0.29
TNF-α	−5.77	−5.28	−6.57
Constant	−63.39	−75.75	−79.11
Prior probability	0.33	0.33	0.33

Unit of all cytokines is pg/mL; prior probability is equal for each cluster in the test study.

**Table E4:** Summary statistics by disease and biological subgroups in the validation study that were identified using linear discriminant analysis

Variable	G1 (asthma in group 1) (n = 94)	G2 (asthma in group 2) (n = 55)	G3 (asthma in group 3) (n = 7)	G4 (COPD in group 1) (n = 12)	G5 (COPD in group 2) (n = 18)	G6 (COPD in group 3) (n = 28)
Males, n (%)	59 (62.8)	27 (49.1)	3 (42.9)	6 (50)	13 (72.2)	22 (78.6)
Current or ex-smokers	25 (26.6)	24 (43.6)	5 (71.4)	12 (100)	18 (100)	28 (100)
Pack-year history∗	7.5 (4.6-12.2)	7.3 (3.7-14.5)	20.2 (15.77-26)	39.4 (26.0-59.7)	38.1 (27.6-52.7)	43.6 (34.0-55.8)
Age (y)†	51 (1.3)	51 (2.1)	52 (5)	66 (3.2)	70.9 (2.2)	69 (1.7)
Duration of disease (y)	18 (15.2-21.7)	20 (15.4-25.4)	23 (15.8-34.2)	4.1 (2.7-6.3)	3 (2.4-4.8)	4 (3.0-5.6)
BMI (kg/m^2^)†	30.5 (0.7)	29.5 (0.9)	27.2 (2.2)	27.7 (3)	27.5 (1.1)	27.0 (1.0)
Exacerbation number of steroids	3 (0.3)	3 (0.4)	4 (0.8)	2 (0.4)	3 (0.5)	3 (0.6)
Maintenance prednisolone dose use, n (%)	54 (57.4)	28 (50.9)	4 (57.1)	0 (0)	2 (11.1)	3 (10.7)
Daily prednisolone dose (mg)‡§	10 (10-15)	10 (6.25-15)	7.5 (5-12.5)	0 (0-0)	7.5 (5-10)	5 (5-7.5)
Daily inhaled corticosteroid dose (μg/d)‡||	1600 (1000-2000)	1600 (1000-2000)	1600 (1000-2000)	2000 (1200-2000)	650 (0-2000)	800 (0-1800)
Pre-FEV_1_/FVC ratio (%)†	70.2 (1.2)	65.6 (2.1)	65.2 (2.3)	52.7 (5.9)	50.4 (2.8)	55.3 (2.0)
Pre-FEV_1_ predicted (%)†	70.0 (2.3)	69.5 (3.1)	73.1 (7.6)	47.5 (5.9)	50.3 (5.6)	63.5 (3.5)
Sputum neutrophil count (%)†	58.1 (3)	69.8 (3)	56.7 (10.4)	66 (7.4)	79.7 (3.6)	58.7 (4.4)
Sputum eosinophil count (%)	6.5 (4.92-8.58)	3.47 (2.64-4.56)	5.52 (3.68-8.27)	5.23 (2.27-12.05)	2.0 (1.53-2.62)	2.9 (1.97-4.21)
TCC (× 10^6^ cells/g sputum)	1.06 (0.82-1.38)	2.96 (2.01-4.38)	1.46 (0.58-3.67)	2.78 (1.56-4.96)	5.6 (2.99-10.51)	3.4 (2.32-5.0)
IL-1B (pg/mL)	53.7 (41.7-69.1)	425.4 (293.3-617.1)	13.5 (2.8-65.8)	57.7 (29.4-113.5)	1010.6 (501-2038.7)	56.4 (36.9-86.3)
IL-5 (pg/mL)	1.3 (1.05-1.7)	1.4 (1.0-2.0)	0.7 (0.5-1.0)	2.3 (1.0-5.3)	0.7 (0.2-1.9)	0.9 (0.5-1.7)
IL-6 (pg/mL)	25.9 (19.5-34.4)	247.4 (193.2-316.9)	205.9 (105.0-403.6)	27.8 (10.7-72.5)	370.2 (177.0-774.0)	391.4 (255.8-598.9)
IL-6R (pg/mL)	197.7 (140.4-278.5)	818.7 (694.1-965.7)	20.6 (1.6-265.7)	115.1 (53.3-248.7)	216.5 (136.3-343.8)	131.1 (98.6-174.4)
IL-8 (pg/mL)	1241 (816-1886)	14,749 (12,142-17,916)	128.9 (5.1-3228.9)	995.2 (359.5-2755.4)	4122.4 (3552.4-4783.7)	2511.4 (1827.5-3451.3)
IL-10 (pg/mL)	2.4 (1.9-2.9)	3.2 (2.2-4.5)	1.7 (1.1-2.7)	0.3 (0.2-0.5)	7.1 (3.3-15.1)	0.4 (0.2-0.7)
CCL-2 (pg/mL)	178.7 (150.8-211.8)	415.4 (321.9-536.1)	415.3 (207.5-831.1)	335.7 (171.5-657.1)	458.5 (273.9-767.6)	769.3 (530.5-1115.6)
CCL-4 (pg/mL)	110.2 (72.9-166.7)	640.5 (499.4-821.4)	24.9 (4.4-142.0)	226.3 (57.8-885.4)	1999.4 (1031.1-3876.8)	1065.2 (669.9-1693.6)
CCL-5 (pg/mL)	4.5 (3.6-5.6)	19.9 (15.6-25.4)	2.4 (0.8-6.9)	4.4 (2.2-8.8)	10.0 (5.8-17.2)	4.2 (2.9-6.2)
CCL-13 (pg/mL)	16.3 (13.7-19.4)	19.2 (14.3-26.0)	10.3 (5.5-19.1)	29.9 (12.0-74.4)	16.1 (9.4-27.4)	56.2 (40.3-78.5)
CCL-17 (pg/mL)	20.7 (15.8-27.1)	30.2 (22.4-40.7)	8.8 (5.3-14.8)	26.4 (9.6-72.5)	14.4 (7.5-27.5)	51.1 (32.8-79.6)
CXCL-10 (pg/mL)	243.6 (168.7-351.7)	1303.4 (898.0-1892.0)	51.2 (5.9-445.3)	183 (72.4-462.1)	221.0 (90.1-542.0)	379.3 (249.6-576.5)
CXCL-11 (pg/mL)	13.4 (9.4-18.7)	57.3 (36.1-90.7)	16.7 (5.3-52.6)	7.0 (1.4-36.0)	8.9 (2.6-30.1)	25.7 (16.4-40.4)
TNF-α (pg/mL)	1.5 (1.2-1.8)	16.7 (11.0-25.6)	1.9 (0.9-4.2)	0.8 (0.4-1.5)	63.9 (37.4-109.1)	1.8 (1.0-3.2)

Variable	Pairwise comparison between asthma across the 3 clusters, *P* value			Pairwise comparison between asthma and COPD overlap in each cluster, *P* value			Pairwise comparison between COPD across the 3 clusters		
	G1 vs G2	G1 vs G3	G2 vs G3	G1 vs G4	G2 vs G5	G3 vs G6	G4 vs G5	G4 vs G6	G5 vs G6
Males, n (%)	.1	.42	.99	.53	.074	.15	.27	.13	.73
Current or ex-smokers	.05	.023	.24	<.0001	<.0001	.035	.99	.99	.99
Pack-year history∗	.97	.16	.2	<.0001	<.0001	.032	.9	.68	.52
Age (y)†	.95	.83	.83	<.0001	<.0001	<.0001	.17	.32	.56
Duration of disease (y)	.57	.48	.66	<.0001	<.0001	<.0001	.54	.97	.43
BMI (kg/m^2^)†	.37	.21	.41	.2	.26	.93	.94	.77	.75
Exacerbation number of steroids	.8	.45	.37	.26	.89	.27	.38	.57	.7
Maintenance prednisolone dose use, n (%)	.44	.99	.99	<.0001	.005	.018	.5	.54	.99
Daily prednisolone dose (mg)‡§	.67	.29	.46	—	.41	.43	—	—	.56
Daily inhaled corticosteroid dose (μg/d)‡||	.93	.45	.42	.58	.023	.1	.12	.02	.75
Pre-FEV_1_/FVC ratio (%)†	.042	.32	.94	<.0001	<.0001	.025	.64	.58	.15
Pre-FEV_1_ predicted (%)†	.9	.72	.69	.001	.003	.23	.72	.033	.042
Sputum neutrophil count (%)†	.01	.91	.21	.35	.077	.86	.094	.33	.001
Sputum eosinophil count (%)	.003	.76	.31	.59	.033	.18	.015	.098	.18
TCC (× 10^6^ cells/g sputum)	<.0001	.6	0.29	.012	.11	.098	.1	.6	.17
IL-1B (pg/mL)	<.0001	.01	<.0001	.85	.03	.02	<.0001	.96	<.0001
IL-5 (pg/mL)	.7	.19	.15	.14	.078	.68	.078	.16	.59
IL-6 (pg/mL)	<.0001	<.0001	.63	.87	.19	.18	<.0001	<.0001	.89
IL-6R (pg/mL)	<.0001	<.0001	<.0001	.29	<.0001	.011	.091	.7	.062
IL-8 (pg/mL)	<.0001	.003	<.0001	.72	<.0001	.001	<.0001	.011	.024
IL-10 (pg/mL)	.12	.45	.23	<.0001	.038	.024	<.0001	.83	<.0001
CCL-2 (pg/mL)	<.0001	.017	.99	.022	.72	.15	.44	.03	.11
CCL-4 (pg/mL)	<.0001	.032	<.0001	.26	<.0001	<.0001	.001	.007	.12
CCL-5 (pg/mL)	<.0001	.12	<.0001	.94	.013	.23	.055	.92	.012
CCL-13 (pg/mL)	.31	.23	.16	.043	.56	<.0001	.15	.12	<.0001
CCL-17 (pg/mL)	.074	.082	.007	.57	.026	.001	.25	.18	.002
CXCL-10 (pg/mL)	<.0001	.024	<.0001	.6	<.0001	.006	.74	.17	.24
CXCL-11 (pg/mL)	<.0001	.73	.08	.26	.001	.43	.77	.084	.071
TNF-α (pg/mL)	<.0001	.59	.001	.042	.002	.9	<.0001	.12	<.0001

Groups 1, 2 and 3 were predicted using linear discriminant analysis. Data presented as geometric mean (95% CI) unless otherwise stated. *BMI*, Body mass index; *FVC*, forced vital capacity; *TCC*, total sputum cell count.

∗ Pack-year history of current and ex-smokers.

† Mean (SEM).

‡ Median (first and third quartiles).

§ Dose for only those subjects prescribed daily prednisolone.

|| Beclomethasonedipropionate equivalent.

**Table E5:** Summary statistics by disease and biological subgroups in the validation data set that were obtained using IL1β cutoff and disease status

Variable	Group 1	Group 2		Group 3	Pairwise comparison, *P* value		
	G1 (asthma in group 1) (n = 103)	G2 (asthma in group 2) (n = 63)	G3 (COPD in group 2) (n = 26)	G4 (COPD in group 3) (n = 32)	G1 vs G2	G2 vs G3	G3 vs G4
Males, n (%)	66 (64.1)	32 (50.8)	19 (73.1)	22 (68.7)	.09	.05	.72
Current or ex-smokers	28 (27.2)	28 (44.4)	26 (100)	32 (100)	.022	<.0001	.99
Pack-year history∗	9.5 (5.8-15.7)	6.9 (4.0-12.0)	40.4 (30.2-54.1)	41.5 (33.5-51.6)	.4	<.0001	.88
Age (y)†	49 (1.2)	52 (2.0)	71 (2.0)	68 (1.6)	.29	<.0001	.18
Duration of disease (y)	17 (14.1-20.7)	21 (17.3-25.7)	4.2 (3.1-5.7)	3.6 (2.7-4.7)	.15	<.0001	.45
BMI (kg/m^2^)†	29.9 (0.7)	29.5 (0.8)	27.7 (0.9)	27.0 (1.3)	.71	.2	.67
Exacerbation number of steroids	3.5 (0.3)	3.1 (0.4)	3.3 (0.6)	2.3 (0.4)	.39	.7	.12
Maintenance prednisolone dose use, n (%)	57 (55.3)	32 (50.8)	3 (11.5)	2 (6.2)	.57	.001	.65
Daily prednisolone dose (mg)‡§	10 (8-15)	10 (5-12.5)	5 (5-10)	6.25 (5-7.5)	.27	.22	.99
Daily inhaled corticosteroid dose (μg/d)‡||	1600 (1000-2000)	2000 (1600-2000)	1000 (0-2000)	900 (200-2000)	.07	.028	.68
Pre-FEV_1_/FVC ratio (%)†	70.1 (1.2)	66.3 (1.9)	49.1 (2.4)	56.6 (2.4)	.069	<.0001	.033
Pre-FEV_1_ predicted (%)†	71.2 (2.3)	69.6 (2.7)	51.0 (4.4)	60.2 (3.6)	.66	<.0001	.11
Sputum neutrophil count (%)†	59.0 (2.8)	67.9 (3.2)	75.9 (3.8)	59.2 (4.2)	.04	.15	.005
Sputum eosinophil count (%)	5.6 (4.3-7.3)	4.4 (3.4-5.8)	2.1 (1.6-2.7)	3.9 (2.5-5.9)	.24	.001	.024
TCC (× 10^6^ cells/g sputum)	0.98 (0.74-1.28)	2.5 (1.8-3.7)	4.8 (3.0-7.8)	3.1 (2.2-4.5)	<.0001	.053	.15
IL-1B (pg/mL)	37.0 (29.1-47.0)	485.8 (364.8-646.8)	642.5 (370.5-1114.2)	40.0 (28.2-56.6)	<.0001	.34	<.0001
IL-5 (pg/mL)	1.2 (1.0-1.5)	1.5 (1.1-2.1)	1.0 (0.4-2.4)	1.0 (0.6-1.7)	.33	.33	.94
IL-6 (pg/mL)	34.7 (25.6-47.0)	149.5 (107.4-207.9)	340.3 (169.5-683.0)	157.7 (88.7-280.2)	<.0001	.02	.098
IL-6R (pg/mL)	153.2 (102.7-228.6)	774.7 (659.6-910.0)	239 (168.2-339.5)	101.7 (74.1-139.5)	<.0001	<.0001	.001
IL-8 (pg/mL)	975.0 (596.9-1592.3)	11958 (9587-14917)	3883 (3164.0-4765.4)	1646.2 (1040.9-2603.3)	<.0001	<.0001	.003
IL-10 (pg/mL)	2.2 (1.8-2.6)	3.2 (2.4-4.4)	2.8 (1.2-6.7)	0.4 (0.2-0.6)	.026	.72	<.0001
CCL-2 (pg/mL)	202.1 (166.8-244.9)	346.5 (279.5-429.6)	641.5 (405.6-1014.8)	488.3 (340.0-701.4)	<.0001	.008	.36
CCL-4 (pg/mL)	101.3 (65.3-157.3)	491.6 (368.5-655.9)	1533.6 (710.3-3311.1)	631.5 (382.8-1041.7)	<.0001	.001	.055
CCL-5 (pg/mL)	5.2 (4.1-6.7)	12.9 (9.6-17.3)	9.1 (6.1-13.7)	3.7 (2.54-5.5)	<.0001	.21	.003
CCL-13 (pg/mL)	14.7 (12.4-17.5)	20.1 (15.4-26.2)	27.3 (15.5-48.3)	39.4 (27.4-56.6)	.046	.28	.28
CCL-17 (pg/mL)	19.5 (15.1-25.0)	26.9 (19.9-36.3)	24.4 (12.7-46.9)	35.7 (22.9-55.6)	.11	.76	.33
CXCL-10 (pg/mL)	250.4 (163.4-383.8)	850.2 (592.4-1220.2)	251.1 (123.0-512.7)	297.7 (194.2-456.6)	<.0001	.001	.68
CXCL-11 (pg/mL)	17.3 (12.4-24.2)	33.5 (21.1-53.2)	12.3 (4.9-30.8)	15.8 (7.8-31.8)	.022	.037	.67
TNF-α (pg/mL)	1.6 (1.3-2.0)	11.9 (7.7-18.5)	22.6 (10.2-50.0)	1.2 (0.7-2.1)	<.0001	.15	<.0001

Data presented as geometric mean (95% CI) unless otherwise stated. *BMI*, Body mass index; *group 1*, pure asthma (IL-1B < 130 pg/mL); *group 2*, asthma and COPD overlap (IL-1B ≥ 130 pg/mL); *group 3*, pure COPD (IL-1B < 130 pg/mL); *FVC*, forced vital capacity; *TCC*, total sputum cell count.

∗ Pack-year history of current and ex-smokers.

† Mean (SEM).

‡ Median (first and third quartiles).

§ Dose for only those subjects prescribed daily prednisolone.

|| Beclomethasonedipropionate equivalent.

**Table I:** Summary statistics across asthma and COPD in the test study

Variable	Asthma (N = 86)	COPD (N = 75)	*P* value
Males, n (%)	43 (50)	53 (70.7)	.008
Current or ex-smokers, n (%)	32 (37.2)	72 (96.0)	<.0001
Pack-year history∗	4.6 (2.98-7.26)	40 (34.46-46.39)	<.0001
Age (y)†	54 (1.3)	69 (1.1)	<.0001
Duration of disease (y)	21 (16.4-26.5)	5 (4.12-6.55)	<.0001
BMI (kg/m^2^)†	30.4 (0.8)	25.7 (0.5)	<.0001
Exacerbation number of steroids	3 (0.23)	4 (0.31)	.007
Maintenance prednisolone dose use, n (%)	52 (60.5)	8 (10.7)	<.0001
Daily prednisolone dose (mg)‡§	10 (7.5-15)	5 (5-5)	.002
Daily inhaled corticosteroid dose (μg/d)‡||	1600 (100-2000)	1200 (800-2000)	.05
Pre-FEV_1_ (L)†	2.15 (0.1)	1.28 (0.1)	<.0001
Pre-FEV_1_/FVC ratio (%)†	67.6 (1.5)	49.8 (1.6)	<.0001
Pre-FEV_1_ predicted (%)†	74.6 (2.4)	45.4 (2.1)	<.0001
Post-FEV_1_ (L)†	2.32 (0.09)	1.32 (0.06)	<.0001
Post-FEV_1_ predicted (%)†	79.8 (2.4)	47.1 (2.1)	<.0001
Sputum neutrophil count (%)†	63.2 (2.5)	69.7 (2.5)	.07
Sputum eosinophil count (%)	2.1 (1.38-3.1)	1.4 (0.98-1.93)	.14
Sputum macrophage count (%)	16.7 (13.41-20.78)	16.2 (13.3-19.8)	.84
TCC (× 10^6^ cells/g sputum)	1.64 (1.28-2.11)	3.34 (2.53-4.41)	<.0001
Blood eosinophil × 10^9^/L	0.23 (0.19-0.29)	0.22 (0.19-0.26)	.63
Blood neutrophil × 10^9^/L†	5.81 (0.2)	5.59 (0.2)	.50
CFU >10^7^/mL or positive culture, n (%)	16 (18.6)	30 (40)	.003
VAS score-cough (mm)†	34 (2.7)	44 (3.4)	.021
VAS score-dyspnea (mm)†	34 (2.8)	46 (3.0)	.004
IL-1β (pg/mL)	70.3 (53-93.1)	73.5 (47.1-114.6)	.86
IL-4 (pg/mL)‡	0.36 (0.36-0.59)	0.36 (0.36-0.36)	.002
Detectable IL-4, n (%)	28 (32.6)	7 (9.3)	<.0001
IL-5 (pg/mL)	2.7 (1.9-4.0)	1.1 (0.8-1.5)	.001
IL-6 (pg/mL)	42.8 (30.5-60.1)	439.2 (320.5-601.8)	<.0001
IL-6R (pg/mL)	243.4 (195.1-303.7)	147.8 (119.3-183.1)	.002
IL-8 (pg/mL)	3118 (2314-4201)	4390 (3372-5715)	.098
IL-10 (pg/mL)	0.73 (0.5-1.1)	1.0 (0.6-1.7)	.27
IL-13 (pg/mL)	8.1 (6.4-10.3)	3.4 (2.5-4.6)	<.0001
IL-17 (pg/mL)‡	2.12 (2.12-5.23)	2.12 (2.12-2.12)	<.001
Detectable IL-17, n (%)	37 (43.0)	2 (2.7)	<.0001
CCL-2 (pg/mL)	284.3 (226.6-356.7)	573.1 (455.3-721.4)	<.0001
CCL-3 (pg/mL)	30.6 (22.9-40.7)	67.1 (53.2-84.7)	<.0001
CCL-4 (pg/mL)	359.5 (247.0-523.3)	958.3 (778.5-1179.7)	<.0001
CCL-5 (pg/mL)	8.7 (6.9-11.0)	3.3 (2.6-4.1)	<.0001
CCL-13 (pg/mL)	19.2 (14.7-25.0)	28.1 (21.3-37.0)	.052
CCL-17 (pg/mL)	25.9 (19.5-34.6)	20.3 (15.1-27.2)	.24
CCL-26 (pg/mL)	9.9 (6.8-14.3)	2.9 (2.0-4.1)	<.0001
CXCL-10 (pg/mL)	726.9 (526-1004.7)	277.9 (205.3-376.3)	<.0001
CXCL-11 (pg/mL)	57.2 (39.7-82.5)	11.6 (7.6-17.9)	<.0001
TNF-α (pg/mL)	3.2 (2.3-4.5)	5.4 (3.3-8.9)	.093
VEGF (pg/mL)	1427 (1214-1678)	1284 (1129-1461)	.33
IFN-γ (pg/mL)‡	0.13 (0.13-0.23)	0.13 (0.13-0.34)	.39
Detectable IFN-γ, n (%)	23 (26.7)	24 (32)	.46

Data presented as geometric mean (95% CI) unless otherwise stated. *BMI*, Body mass index; *CFU*, colony-forming units; *FVC*, forced vital capacity; *TCC*, total sputum cell count; *VAS*, visual analog scale; *VEGF*, vascular endothelial growth factor.

∗ Pack-year history of current and ex-smokers.

† Mean (SEM).

‡ Median (first and third quartiles).

§ Dose for only those subjects prescribed daily prednisolone.

|| Beclomethasonedipropionate equivalent.

**Table II:** Summary statistics across the 3 identified biological clusters in the test study

Variable	Cluster 1	Cluster 2	Cluster 3	Pairwise comparison, *P* value			ANOVA, *P* value
	Asthma (n = 55); COPD (n = 3)	Asthma (n = 28); COPD (n = 19)	Asthma (n = 2); COPD (n = 39)	C1 vs C2	C1 vs C3	C2 vs C3	
Males, n (%)	32 (55.2)	26 (55.3)	28 (68.3)	.99	.19	.21	.35
Current or ex-smokers, n (%)	22 (37.9)	29 (61.7)	40 (97.6)	.015	<.0001	<.0001	<.0001
Pack-year history∗	6.6 (3.8-11.6)	11.0 (6.1-19.9)	40.3 (33.6-48.3)	.13	<.0001	<.0001	<.0001
Age (y)†	55 (1.5)	60 (2.1)	67 (1.8)	.038	<.0001	.008	<.0001
Duration of disease (y)	23 (17.2-29.6)	9 (5.8-12.8)	6 (4.0-7.9)	<.0001	<.0001	.12	<.0001
BMI (kg/m^2^)†	30.2 (1.0)	28.8 (1.0)	25.3 (0.7)	.28	<.0001	.005	.001
Exacerbation number of steroids	3 (0.3)	3 (0.3)	4 (0.4)	.31	.002	.047	.007
Maintenance prednisolone dose use, n (%)	34 (58.6)	19 (40.4)	6 (14.6)	.06	<.0001	.001	<.0001
Daily prednisolone dose (mg)‡§	10 (10-15)	7.5 (5-10)	5 (5-7.5)	.008	.006	.5	.003
Daily inhaled corticosteroid dose (μg/d)‡||	1800 (1000-2000)	1000 (800-2000)	1000 (800-2000)	.024	.28	.4	.08
Pre-FEV_1_ (L)†	2.19 (0.1)	1.74 (0.1)	1.3 (0.1)	.002	<.0001	.006	<.0001
Pre-FEV_1_/FVC ratio (%)†	69.0 (1.9)	58.5 (2.3)	49.7 (2.4)	<.0001	<.0001	.011	<.0001
Pre FEV_1_ predicted (%)†	77.0 (2.7)	59.9 (3.7)	47.0 (3)	<.0001	<.0001	.01	<.0001
Post-FEV_1_ (L)†	2.35 (0.1)	1.88 (0.13)	1.37 (0.09)	.005	<.0001	.003	<.0001
Post-FEV_1_ predicted (%)†	81.7 (2.7)	63.9 (3.9)	49.1 (3)	<.0001	<.0001	.005	<.0001
Sputum eosinophil count (%)	3.9 (2.4-6.4)	0.7 (0.5-0.9)	2.0 (1.25-3.17)	<.0001	.039	<.0001	<.0001
Sputum neutrophil count (%)†	58.8 (3.1)	77.18 (3)	59.1 (3.1)	<.0001	.95	<.0001	<.0001
Sputum macrophage count (%)	16.6 (12.5-21.9)	12.2 (9.2-16.1)	25.7 (21.24-31.07)	.1	.026	<.0001	.002
TCC (× 10^6^ cells/g sputum)	1.31 (1.0-1.8)	4.6 (3.3-6.4)	1.8 (1.3-2.6)	<.0001	.15	<.0001	<.0001
Blood eosinophil × 10^9^/L	0.24 (0.18-0.32)	0.25 (0.2-0.32)	0.21 (0.16-0.27)	.73	.54	.28	.66
Blood neutrophil × 10^9^/L†	5.74 (0.3)	5.82 (0.3)	5.77 (0.4)	.85	.94	.92	.98
CFU >10^7^/mL or positive culture, n (%)	8 (13.8)	26 (55.3)	9 (21.9)	<.0001	.29	.001	<.0001
VAS score-cough (mm)†	30 (3.0)	48 (4.0)	36 (4.4)	.001	.24	.052	.002
VAS score-dyspnea (mm)†	31 (3.5)	46 (3.3)	46 (4.5)	.003	.006	.93	.0036
IL-1β (pg/mL)	39.5 (30.8-50.8)	379.5 (257.3-559.8)	23.5 (17.2-32.2)	<.0001	.025	<.0001	<.0001
IL-4 (pg/mL)‡	0.36 (0.36-0.59)	0.36 (0.36-0.59.)	0.36 (0.36-0.36)	.58	.013	.07	.004
Detectable IL-4, n (%)	20 (34.5)	13 (27.7)	2 (4.9)	.45	<.0001	.005	.001
IL-5 (pg/mL)	2.6 (1.6-4.2)	2.2 (1.4-3.4)	1.4 (0.9-2.2)	.56	.083	.22	.22
IL-6 (pg/mL)	21.3 (15-30.4)	271.4 (192.2-383.3)	486.2 (327.7-721.4)	<.0001	<.0001	.031	<.0001
IL-6R (pg/mL)	163.2 (126.0-211.6)	433.4 (344.2-545.6)	112.4 (88.6-142.6)	<.0001	.04	<.0001	<.0001
IL-8 (pg/mL)	1658 (1205-2280)	10884 (8709-13603)	3059 (2209-4236)	<.0001	.005	<.0001	<.0001
IL-10 (pg/mL)	0.33 (0.25-0.45)	5.5 (3.5-8.7)	0.34 (0.2-0.6)	<.0001	.89	<.0001	<.0001
IL-13 (pg/mL)	10.4 (7.7-14.0)	4.8 (3.8-6.2)	3.5 (2.4-5.2)	.001	<.0001	.18	<.0001
IL-17 (pg/mL)‡	2.12 (2.12-3.97)	2.12 (2.12-5.85)	2.12 (2.12-2.12)	.48	.003	.006	.002
Detectable IL-17, n (%)	20 (34.5)	19 (40.4)	0 (0.0)	.53	<.0001	<.0001	<.0001
CCL-2 (pg/mL)	209.8 (168.3-261.5)	495.4 (378.1-649.1)	764.5 (538.8-1084.7)	<.0001	<.0001	.055	<.0001
CCL-3 (pg/mL)	20.2 (14.9-27.4)	97.4 (71.6-132.6)	47.9 (35.7-64.1)	<.0001	<.0001	.002	<.0001
CCL-4 (pg/mL)	237.8 (147.1-384.3)	1138.3 (847.8-1528.3)	807 (614.1-1060.5)	<.0001	<.0001	.1	<.0001
CCL-5 (pg/mL)	5.6 (4.5-7.0)	14.9 (11.1-20.1)	2.2 (1.8-2.8)	<.0001	<.0001	<.0001	<.0001
CCL-13 (pg/mL)	18.1 (12.9-25.5)	18.9 (13.6-26.2)	43.2 (32.6-57.2)	.86	<.0001	<.0001	<.001
CCL-17 (pg/mL)	27 (19.2-37.9)	20.5 (14.0-30.0)	30.8 (21.5-44.2)	.28	.61	.13	.31
CCL-26 (pg/mL)	12.4 (7.8-19.9)	5.0 (3.4-7.5)	2.9 (1.9-4.6)	.004	<.0001	.081	<.001
CXCL-10 (pg/mL)	418.7 (286.9-611.1)	860.1 (534.1-1384.9)	381.8 (262.5-555.3)	.014	.76	.012	.016
CXCL-11 (pg/mL)	34.1 (22.8-51.0)	42.5 (20.1-89.6)	19.2 (12.3-30.0)	.56	.15	.089	.15
TNF-α (pg/mL)	1.4 (1.1-1.9)	29.9 (19.5-45.9)	1.7 (1.1-2.5)	<.0001	.62	<.0001	<.0001
VEGF (pg/mL)	1020 (858-1213)	2199 (1871-2584)	1237 (1040-1471)	<.0001	.12	<.0001	<.0001
IFN-γ (pg/mL)‡	0.13 (0.13-0.13)	0.23 (0.13-1.7)	0.13 (0.13-0.14)	<.001	.4	.012	.001
Detectable IFN-γ, n (%)	11 (19)	24 (51.1)	11 (26.8)	.001	.35	.02	.002

Data presented as geometric mean (95% CI) unless otherwise stated. *BMI*, Body mass index; *C*, cluster; *CFU*, colony-forming units; *FVC*, forced vital capacity; *TCC*, total sputum cell count; *VAS*, visual analog scale; *VEGF*, vascular endothelial growth factor.

∗ Pack-year history of current and ex-smokers.

† Mean (SEM).

‡ Median (first and third quartiles).

§ Dose for only those subjects prescribed daily prednisolone.

|| Beclomethasonedipropionate equivalent.

**Table III:** Summary statistics across the validation subgroups that were identified using linear discriminant analysis

Variable	Group 1	Group 2	Group 3	Pairwise comparison, *P* value			ANOVA, *P* value
	Asthma (n = 94); COPD (n = 12)	Asthma (n = 55); COPD (n = 18)	Asthma (n = 7); COPD (n = 28)	G1 vs G2	G1 vs G3	G2 vs G3	
Males, n (%)	65 (61.3)	40 (54.8)	25 (71.4)	.38	.28	.1	.25
Current or ex-smokers	37 (34.9)	42 (57.5)	33 (94.3)	.003	<.0001	<.0001	<.0001
Pack-year history∗	13.2 (8.5-20.7)	16.0 (10.0-25.6)	39.6 (31.3-50.1)	.52	<.0001	.002	<.001
Age (y)†	53 (1.3)	56 (2.0)	66 (2.0)	.15	<.0001	.002	<.0001
Duration of disease (y)	15 (12.9-18.7)	13 (9.8-16.8)	6 (4.2-8.5)	.24	<.0001	.002	<.001
BMI (kg/m^2^)†	30.2 (0.7)	29.0 (0.8)	27.1 (0.9)	.22	.017	.13	.05
Exacerbation number of steroids	3 (0.3)	3 (0.3)	3 (0.5)	.94	.82	.87	.97
Maintenance prednisolone dose use, n (%)	54 (50.9)	30 (41.1)	7 (20.0)	.19	.001	.03	.006
Daily prednisolone dose (mg)‡§	10 (10-15)	10 (5-15)	5 (5-10)	.52	.03	.12	.11
Daily inhaled corticosteroid dose (μg/d)‡||	2000 (1000-2000)	1600 (1000-2000)	1000 (200-2000)	.2	<.001	.008	<.001
Pre-FEV_1_/FVC ratio (%)†	68.2 (1.4)	61.8 (1.9)	57.3 (1.8)	.004	<.0001	.14	<.001
Pre-FEV_1_ predicted (%)†	67.6 (2.2)	64.7 (2.9)	65.5 (3.2)	.4	.65	.85	.69
Sputum neutrophil count (%)†	59.1 (2.8)	72.3 (2.5)	58.4 (4.0)	.001	.88	.003	.001
Sputum eosinophil count (%)	6.3 (4.9-8.2)	3.0 (2.4-3.8)	3.2 (2.3-4.4)	<.0001	.003	.79	<.001
TCC (× 10^6^ cells/g sputum)	1.2 (0.9-1.5)	3.5 (2.5-4.8)	2.99 (2.07-4.3)	<.0001	.001	.6	<.0001
IL-1B (pg/mL)	54.1 (42.7-68.5)	526.6 (375.5-738.4)	42.4 (26-69.2)	<.0001	.36	<.0001	<.0001
IL-5 (pg/mL)	1.4 (1.1-1.8)	1.2 (0.8-1.7)	0.9 (0.5-1.5)	.43	.085	.34	.22
IL-6 (pg/mL)	26.1 (19.9-34.3)	273.3 (210.6-354.6)	344.2 (237.2-499.3)	<.0001	<.0001	.32	<.0001
IL-6R (pg/mL)	186.0 (135.5-255.3)	589.8 (476.6-729.8)	90.5 (50.3-162.8)	<.0001	.013	<.0001	<.0001
IL-8 (pg/mL)	1210 (822-1783)	10,771 (8,846-13,115)	1387 (643-2989)	<.0001	.7	<.0001	<.0001
IL-10 (pg/mL)	1.9 (1.5-2.4)	3.9 (2.8-5.4)	0.5 (0.3-0.9)	.001	<.0001	<.0001	<.0001
CCL-2 (pg/mL)	191.9 (161.7-227.8)	425.6 (338.6-535.0)	680.1 (486.9-949.8)	<.0001	<.0001	.025	<.0001
CCL-4 (pg/mL)	119.6 (80.3-178.1)	848.1 (646.8-1111.9)	502.5 (248-1018.2)	<.0001	<.0001	.1	<.0001
CCL-5 (pg/mL)	4.5 (3.7-5.6)	16.8 (13.3-21.2)	3.8 (2.6-5.5)	<.0001	.39	<.0001	<.0001
CCL-13 (pg/mL)	17.4 (14.5-21.0)	18.4 (14.2-23.9)	40.0 (27.7-57.9)	.73	<.0001	.001	<.001
CCL-17 (pg/mL)	21.3 (16.4-27.7)	25.2 (19.0 -33.4)	36.0 (23.3-55.6)	.4	.043	.17	.12
CXCL-10 (pg/mL)	235.8 (167.7-331.7)	841.5 (566.5-1249.9)	254.1 (140.7-458.8)	<.0001	.83	.001	<.0001
CXCL-11 (pg/mL)	12.4 (8.7-17.6)	36.1 (22.1-59.1)	23.6 (15.4-35.9)	<.0001	.08	.28	<.001
TNF-α (pg/mL)	1.4 (1.2-1.7)	23.3 (16.1-33.7)	1.8 (1.1-3.0)	<.0001	.32	<.0001	<.0001

Groups 1, 2 and 3 were predicted using linear discriminant analysis. Data presented as geometric mean (95% CI) unless otherwise stated. *BMI*, Body mass index; *FVC*, forced vital capacity; *G*, group.

∗ Pack-year history of current and ex-smokers.

† Mean (SEM).

‡ Median (first and third quartiles).

§ Dose for only those subjects prescribed daily prednisolone.

|| Beclomethasonedipropionate equivalent.

**Table IV:** Summary statistics across the validation subgroups that were identified using IL-1β cutoff and subject disease status (asthma or COPD)

Variable	Group 1	Group 2	Group 3	Pairwise comparison, *P* value			ANOVA, *P* value
	Asthma (n = 103)	Asthma (n = 63); COPD (n = 26)	COPD (n = 32)	G1 vs G2	G1 vs G3	G2 vs G3	
Males, n (%)	66 (64.1)	51 (57.3)	22 (68.7)	.34	.63	.26	.44
Current or ex-smokers	28 (27.2)	54 (60.7)	32 (100)	<.0001	<.0001	<.0001	<.0001
Pack-year history∗	9.5 (5.8-15.7)	17.0 (11.5 -25.2)	41.5 (33.5-51.6)	.06	<.0001	.001	<.001
Age (y)†	49 (1.2)	57 (1.8)	68 (1.6)	<.0001	<.0001	.001	<.0001
Duration of disease (y)	17 (14.1-20.7)	13 (10.6-16.6)	4 (2.7-4.7)	.08	<.0001	<.0001	<.0001
BMI (kg/m^2^)†	29.9 (0.7)	29.0 (0.6)	27 (1.3)	.33	.03	.13	.09
Exacerbation number of steroids	3 (0.3)	3 (0.3)	2 (0.4)	.41	.05	.13	.14
Maintenance prednisolone dose use, n (%)	57 (55.3)	35 (39.3)	2 (6.25)	.027	<.0001	<.0001	<.0001
Daily prednisolone dose (mg)‡§	10 (8-15)	10 (5-10)	6.25 (5-7.5)	.15	.11	.24	.12
Daily inhaled corticosteroid dose (μg/d)‡||	1600 (1000-2000)	2000 (1000-2000)	900 (200-2000)	.52	.006	.008	.13
Pre-FEV_1_/FVC ratio (%)†	70.1 (1.2)	61.0 (1.7)	56.6 (2.4)	<.0001	<.0001	.16	<.0001
Pre-FEV_1_ predicted (%)†	71.2 (2.3)	64.1 (2.5)	60.2 (3.6)	.033	.019	.4	.022
Sputum neutrophil count (%)†	59.0 (2.8)	70.4 (2.5)	59.2 (4.2)	.003	.97	.023	.006
Sputum eosinophil count (%)	5.6 (4.3-7.3)	3.5 (2.8-4.4)	3.9 (2.5-5.9)	.009	.12	.68	.028
TCC (× 10^6^ cells/g sputum)	0.98 (0.7-1.3)	3.1 (2.3-4.1)	3.1 (2.2-4.5)	<.0001	<.0001	.97	<.0001
IL-1B (pg/mL)	37.0 (29.1-47)	527.1 (407.1-682.5)	40.0 (28.2-56.6)	<.0001	.75	<.0001	<.0001
IL-5 (pg/mL)	1.2 (1.0 -1.5)	1.3 (1.0 -1.9)	1.0 (0.6-1.7)	.69	.45	.37	.59
IL-6 (pg/mL)	34.7 (25.6-47)	190 (138.4-261)	157.7 (88.8-280.2)	<.0001	<.0001	.56	<.0001
IL-6R (pg/mL)	153 (102.7-228.6)	549.4 (454.9-663.6)	101.7 (74.1-139.5)	<.0001	.17	<.0001	<.0001
IL-8 (pg/mL)	975 (597-1592)	8,609 (7,062- 10,496)	1646 (1041-2603)	<.0001	.15	<.0001	<.0001
IL-10 (pg/mL)	2.2 (1.8-2.6)	3.1 (2.2-4.3)	0.4 (0.2-0.6)	.063	<.0001	<.0001	<.0001
CCL-2 (pg/mL)	202 (166.8-244.9)	414.8 (336.4-511.6)	488.3 (340.0-701.4)	<.0001	<.0001	.44	<.0001
CCL-4 (pg/mL)	101.3 (65.3-157.3)	685.4 (497.8-943.8)	631.5 (382.8-1041.7)	<.0001	<.0001	.79	<.0001
CCL-5 (pg/mL)	5.2 (4.1-6.7)	11.7 (9.2-14.8)	3.7 (2.5-5.5)	<.0001	.16	<.0001	<.0001
CCL-13 (pg/mL)	14.7 (12.4-17.5)	22.0 (17.1-28.3)	39.4 (27.4-56.6)	.01	<.0001	.017	<.0001
CCL-17 (pg/mL)	19.5 (15.1-25)	26.1 (19.7-34.7)	35.7 (22.9-55.6)	.13	.025	.26	.057
CXCL-10 (pg/mL)	250.5 (163-384)	595.4 (420.6-842.9)	297.7 (194.2-456.6)	.002	.64	.034	.0052
CXCL-11 (pg/mL)	17.3 (12.4-24.2)	25.0 (16.3-38.5)	15.8 (7.8-31.8)	.18	.81	.28	.32
TNF-α (pg/mL)	1.6 (1.3-2.0)	14.4 (9.8-21.2)	1.3 (0.7-2.1)	<.0001	.39	<.0001	<.0001

Data presented as geometric mean (95% CI) unless otherwise stated. *BMI*, Body mass index; *G*, group; *group 1*, pure asthma (IL-1B < 130 pg/mL); *group 2*, asthma and COPD overlap (IL-1B ≥ 130 pg/mL); *group 3*, pure COPD (IL-1B < 130 pg/mL); *FVC*, forced vital capacity; *TCC*, total sputum cell count.

∗ Pack-year history of current and ex-smokers.

† Mean (SEM).

‡ Median (first and third quartiles).

§ Dose for only those subjects prescribed daily prednisolone.

|| Beclomethasonedipropionate equivalent.

## Statistical methods

Statistical analyses were undertaken by Ghebre and verified independently by Newby. Unsupervised multivariate modeling using factor analysis, principal factor, with orthogonal varimax rotation, was performed to obtain a set of low-dimensional independent and interpretable factors. Sampling adequacy for factor analysis was assessed using Kaiser-Meyer-Olkin.^E1^ Factors were retained on the basis of screeplot (factors above the break in the curve) and eigenvalue above 1. Inflammatory mediators that have high collinearity^E2^ were excluded from factor analysis to avoid multicollinearity; in addition, cytokines that have less than 50% communality,^E3^ total variance explained by the factors, were excluded from the analysis. Because CXCL10 and 11 were highly correlated, and CXCL11's variance was better explained by the factors explored than was CXCL10's variance, CXCL10 was excluded from the model. More than 50% of the subjects had undetectable concentrations of IL-4, IL-17, and IFN-γ. Their distributions were positively skewed and their total variance was not explained by the factors by more than 50%; therefore, they were not included in the factor and cluster analyses. Likewise, because IL-10 levels in more than one-third of asthmatic patients were below the limit of detection, but the concentrations were not different between asthma and COPD then, to avoid bias toward one disease, it was excluded from the model. No similar bias was observed for other inflammatory mediators. Fifteen subjects (1 with asthma and 14 with COPD) who did not have complete record of the cytokine panel were excluded from the biological factor and cluster analysis. Factor scores were calculated for each subject using standardized values and inverse of correlation matrix of the original variables (inflammatory mediators), and factor loadings (Table E1). These scores represent the subjects' predicted value for each factor and retain the relationship between factors, and were used for constructing clusters.^E4^ Although it was possible to identify the underlying structure of the inflammatory mediators using factor analysis, it was impossible to classify subjects into subgroups who have the same underlying structure using factor analysis. Therefore, “k-means cluster analysis on factor scores” approach was used to identify subjects' subgroups that have similar biological profiles. Squared Euclidian distance was used as a measure of similarity. The use of these 2 approaches (cluster and factor analyses) provides a dimensional view of both cytokines and subgroups and was used to explore the unobserved subjects heterogeneity: the factor analysis to capture the profiles of the cytokines and k-means cluster analysis to allow for the classification of individuals into subgroups. The optimal number of clusters was chosen on the basis of screeplot (clusters above the break in the curve) by plotting within cluster sum of the squares against a series of sequential number of clusters^E5^ and by assessing how natural the clusters look on their clinical and biological implications (phenotypes) and clinical meanings and interpretability.

Linear discriminant analysis was performed on the cytokines across the clusters to validate how the identified clusters from the factor scores can be predicted using cytokines measurements, and to identify the contribution of each cytokine in discriminating the clusters. Then, discriminant scores (1 less than the number of clusters) for each subject were calculated using the discriminant functions of a cytokine and the original cytokine values for each subject, and used to represent the subjects' biological cluster membership graphically.

Independent patients with severe asthma and COPD were used for validation. Because the number of cytokines in the validation studies is smaller than the number of cytokines used to identify subgroups in the test study, clusters in the test study were predicted using only those cytokines that existed in the validation group. The betas for each cytokine in each cluster are depicted in Table E3. The classification model for each cluster was developed from the betas for each cytokine in the test study and was subsequently applied in the validation study to assign subjects to subgroups on the basis of the highest discriminant score.

The model fit was as follows:

$$D_{ij}=\beta_{j}+\beta_{j1}X_{1i}+\beta_{j2}X_{2i}+\cdots +\beta_{jk}X_{ki}+\log\left(P_{j}\right)$$

where *D*_*ij*_ is the discriminant score for subject *i* in group *j*, *β*_*j*_ is a constant for the *j*th group, *β*_*jk*_ is the weight (coefficient) for the variable (cytokine) *K* in group *j*, *X*_*ki*_ is the observed value of subject *i* on the *k*th variable; and log (*P*_*j*_) is logarithm of prior probability of group *j* membership.

In addition, classification and regression trees analysis using the rpart R package^E6^ was performed sequentially to all cytokines in the test study, which had high discriminant function, to identify clinically relevant cutoff points. Subsequently, the best determined cytokine cutoff (with the highest sensitivity ratio in discriminating the clusters), together with the disease classification (asthma or COPD), was applied to classify the validation study into subgroups.

Parametric data were expressed as mean with SEM, and log transformed data were presented as geometric mean with 95% CI. The χ^2^ test or the Fisher exact test was used to compare proportions, and 1-way ANOVA was used to compare multiple groups; nonparametric data were presented as median with first and third quartiles and Kruskal-Wallis test was used to compare subgroups. A *P* value of less than .05 was taken as statistically significant. All statistical analyses were performed using STATA/IC version 13.0 for Windows (StataCorp) and R version 2.15.1 (R Foundation for Statistical Computing).
